# Effects of PMMA and Cross-Linked Dextran Filler for Soft Tissue Augmentation in Rats

**DOI:** 10.3390/ijms161226112

**Published:** 2015-12-01

**Authors:** Jung-Bo Huh, Joo-Hyun Kim, Soyun Kim, So-Hyoun Lee, Kyung Mi Shim, Se Eun Kim, Seong Soo Kang, Chang-Mo Jeong

**Affiliations:** 1 Department of Prosthodontics, Dental Research Institute, School of Dentistry, Pusan National University, YangSan 676-870, Korea; identi97@hanmail.net (J.-H.K.); romilove7@hanmail.net (S.-H.L.); 2 School of Dentistry, Pusan National University, YangSan 676-870, Korea; annasoyunkim@gmail.com; 3 Department of Veterinary Surgery, College of Veterinary Medicine, Chonnam National University, Gwangju 500-757, Korea; simchung-98@hanmail.net (K.M.S.); sen0223@gmail.com (S.E.K.)

**Keywords:** biocompatibility, efficacy, PMMA, hydroxypropyl methylcellulose, hyaluronic acid, filler, oral submucosa, cranial subcutaneous, rat

## Abstract

This study was conducted for evaluation of the ability to maintain efficacy and biocompatibility of cross-linked dextran in hydroxypropyl methylcellulose (DiHM) and cross-linked dextran mixed with PMMA in hydroxypropyl methylcellulose (PDiHM), compared with hyaluronic acid (HA) filler. Saline and HA solution was administered in the negative and positive control groups, and DiHM and PDiHM were administered in the test groups (*n* = 10 in each group). The site of cranial subcutaneous injection was the mid-point of the interpupillary line, and the site of intraoral submucosal injection was the ridge crest 2 mm below the cervical line of the mandibular left incisor. Before and immediately after filler injection, intraoral photos and lateral cephalometric radiographs were taken for analysis and comparison of the effect of the filler on the injection sites. The filler injected areas were converted into sequential size changes (%) of the baseline. Histomorphologic examination was performed after 12 weeks. The smallest value in the filler injected area was observed during the experimental period in the normal saline group (*p* < 0.001), which was almost absorbed at 4 weeks (7.19% ± 12.72%). The HA group exhibited a steady decrease in sequential size and showed a lower value than the DiHM and PDiHM groups (saline < HA < DHiM, PDHiM, *p* < 0.001). DiHM and PDiHM tended to increase for the first 4 weeks and later decreased until 12 weeks. In this study on DiHM and PDiHM, there was no histological abnormality in cranial skin and oral mucosa. DiHM and PDiHM filler materials with injection system provide an excellent alternative surgical method for use in oral and craniofacial fields.

## 1. Introduction

Soft tissue augmentation is required for the functional and esthetic recovery of damaged, absorbed, or deformed tissue. Various methods for recovery of such tissue defects have been introduced [1,2,3,4,5,6,7,8]. Among these methods, soft tissue augmentation methods, such as subepithelial connective tissue graft or free gingival graft, use autogenous soft tissue to prevent immune rejection response and inflammation [9]. However, limited soft tissue volume of donor site, patient’s resistance, discomfort, possibility of graft failure, and lack of angiogenesis due to various amounts of fat tissue included could cause problems [10]. To solve these problems, in some cases use of an injection method using commercially available filler material has been reported [11,12,13]. To date, assessment of the filler in evaluation of the cranial subcutaneous area can be worthwhile, but mainly in medical study [14,15]. In recent years, use of a filler for aesthetic or therapeutic purposes in the oral dental gum side has been attempted [11]. However, no animal model of the dental area is associated with use of a filler. Therefore, the experimental part of this study included the oral submucosa area.

Hyaluronic acid fillers are currently used for their good biocompatibility, however their short durability is a problem [14,15,16,17,18,19,20,21]. To achieve longer-lasting effect of fillers, cross-linked dextran-based and PMMA mixed novel materials have recently been developed [22,23]. Cross-linked dextran can stimulate fibroblasts to produce collagen fibers in connective tissue [24,25,26,27]. However, no valid clinical research studies of intraoral application have been reported so far. Accordingly, the aim of this study is to perform research on maintaining efficacy and biocompatibility of cross-linked dextran in hydroxypropyl methylcellulose (DiHM) and cross-linked dextran mixed with PMMA in hydroxypropyl methylcellulose (PDiHM), compared with hyaluronic acid filler through oral submucosa and cranial subcutaneous injection in experimental rats.

## 2. Results

The rats did not develop systemic complications and remained healthy during the entire experimental period, except two rats who died due to technical failure after induction of general anesthesia via intraperitoneal injection. The total number of rats in the experimental group was *n* = 38.

### 2.1. Photographic Measurements of the Filler Injection Area in Oral Submucosa

**Figure 1:**
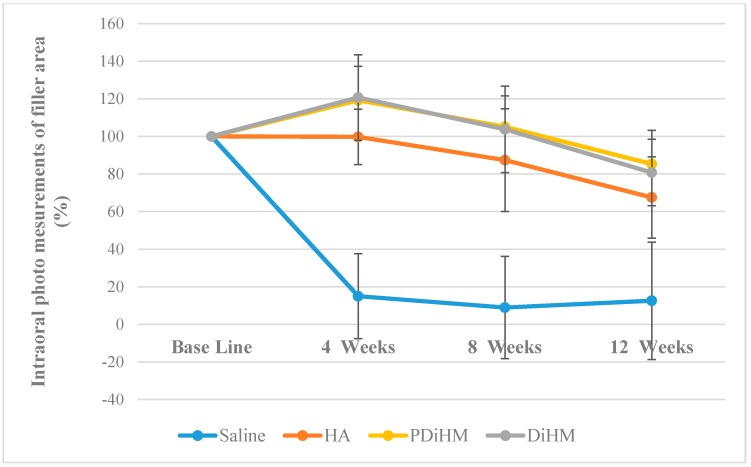
Intraoral photo measurements of the filler area. Values are expressed as mean ± standard deviation of sequential size changes (%) of saline, HA, DiHM, and PDiHM. hyaluronic acid (HA), cross-linked dextran in hydroxypropyl methylcellulose (DiHM), cross-linked dextran mixed with PMMA in hydroxypropyl methylcellulose (PDiHM).

A similar absorption pattern was observed with cranial subcutaneous injection (Saline < HA < DiHM, PDiHM, *p* < 0.001). Sequential size changes from base line to 12 weeks were statistically significant. The saline group showed the smallest value among the groups (*p* < 0.001) and was almost absorbed at 4 weeks (14.96% ± 22.60%). The DiHM, PDiHM groups showed significantly larger filler injection areas than the saline and HA group during the experimental period. The HA group showed a steady decrease, but DiHM and PDiHM tended to increase for the first 4 weeks and later decreased until 12 weeks (*p* < 0.001, Table 1, Figure 1).

**Table 1:** Mean and standard deviation of sequential size changes of the photographic examination area.

Area (%)	Group	*n*	Mean	SD	F (*p*)	Duncan	Time F (*p*)	Interaction Effect F (*p*)	Group Duncan
Base line	Saline	9	100.00	-	-	-	39.636 *** (0.000)	17.968 *** (0.000)	Saline < HA < DiHM, PDiHM
	HA	10	100.00	-					
	DiHM	10	100.00	-					
	PDiHM	9	100.00	-					
4-week	Saline	9	14.96	22.60	58.577 *** (0.000)	Saline < HA < DiHM, PDiHM			
	HA	10	99.75	14.70					
	DiHM	10	120.70	22.74					
	PDiHM	9	119.22	18.08					
8-week	Saline	9	8.93	27.24	33.025 *** (0.000)	Saline < HA DiHM, PDiHM			
	HA	10	87.39	27.32					
	DiHM	10	103.78	23.02					
	PDiHM	9	105.12	16.42					
12-week	Saline	9	12.55	31.22	20.025 *** (0.000)	Saline < HA DiHM, PDiHM			
	HA	10	67.53	21.62					
	DiHM	10	80.79	17.70					
	PDiHM	9	85.36	17.88					

*** *p* < 0.001, the numbers in parentheses are *p* values. Hyaluronic acid (HA), cross-linked dextran in hydroxypropyl methylcellulose (DiHM), cross-linked dextran mixed with PMMA in hydroxypropyl methylcellulose (PDiHM).

### 2.2. Radiographic Measurements of the Filler Injection Area in Cranial Subcutaneous Tissue

**Figure 2:**
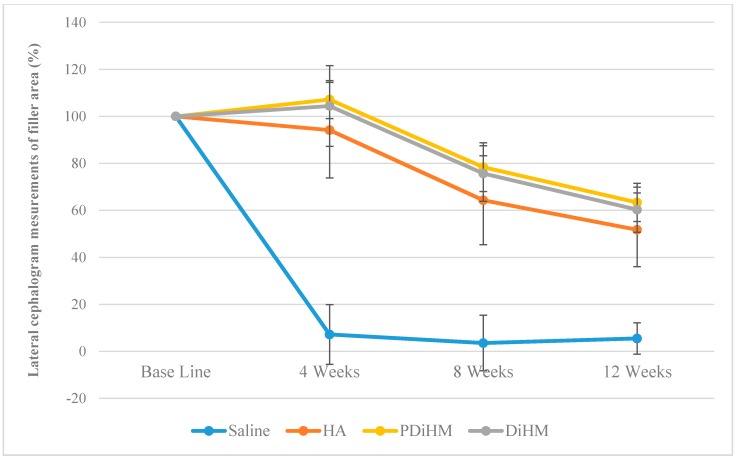
Lateral cephalometric radiograph measurements of the filler area. Values are expressed as mean ± standard deviation of sequential size changes (%) of saline, HA, DiHM, and PDiHM. hyaluronic acid (HA), cross-linked dextran in hydroxypropyl methylcellulose (DiHM), cross-linked dextran mixed with PMMA in hydroxypropyl methylcellulose (PDiHM).

The filler injected areas were converted into sequential size changes (%) of the baseline. The smallest value was observed during the experimental period in the normal saline group (*p* < 0.001), which was almost absorbed at 4 weeks (7.19% ± 12.72%). The HA group exhibited a steady decrease in sequential size and showed a lower value compared with the DiHM and PDiHM groups (saline < HA < DHiM, PDHiM, *p* < 0.001). DiHM and PDiHM tended to increase for the first 4 weeks and later decreased until 12 weeks. No statistical difference was observed between the DiHM and PDiHM groups (Table 2; Figure 2).

**Table 2:** Mean and standard deviation of sequential size changes of the radiographic examination area.

Area (%)	Group	*n*	Mean	SD	F (*p*)	Duncan	Time F (*p*)	Interaction Effect F (*p*)	Group Duncan
Base line	Saline	9	100.00	-	-	-	207.702 *** (0.000)	34.243 *** (0.000)	Saline < HA < DiHM, PDiHM
	HA	10	100.00	-					
	DiHM	10	100.00	-					
	PDiHM	9	100.00	-					
4-week	Saline	9	7.19	12.72	86.048 *** (0.000)	Saline < HA, DiHM, PDiHM			
	HA	10	94.16	20.33					
	DiHM	10	104.43	17.21					
	PDiHM	9	107.17	8.08					
8-week	Saline	9	3.53	11.83	59.429 *** (0.000)	Saline < HA, DiHM < PDiHM			
	HA	10	64.31	18.93					
	DiHM	10	75.69	11.80					
	PDiHM	9	78.40	10.35					
12-week	Saline	9	5.49	6.69	57.097 *** (0.000)	Saline < HA, DiHM < PDiHM			
	HA	10	51.75	15.69					
	DiHM	10	60.30	9.63					
	PDiHM	9	63.37	8.13					

*** *p* < 0.001, the numbers in parentheses are *p* values. Hyaluronic acid (HA), cross-linked dextran in hydroxypropyl methylcellulose (DiHM), cross-linked dextran mixed with PMMA in hydroxypropyl methylcellulose (PDiHM).

### 2.3. Histomorphologic Examination

**Figure 3:**
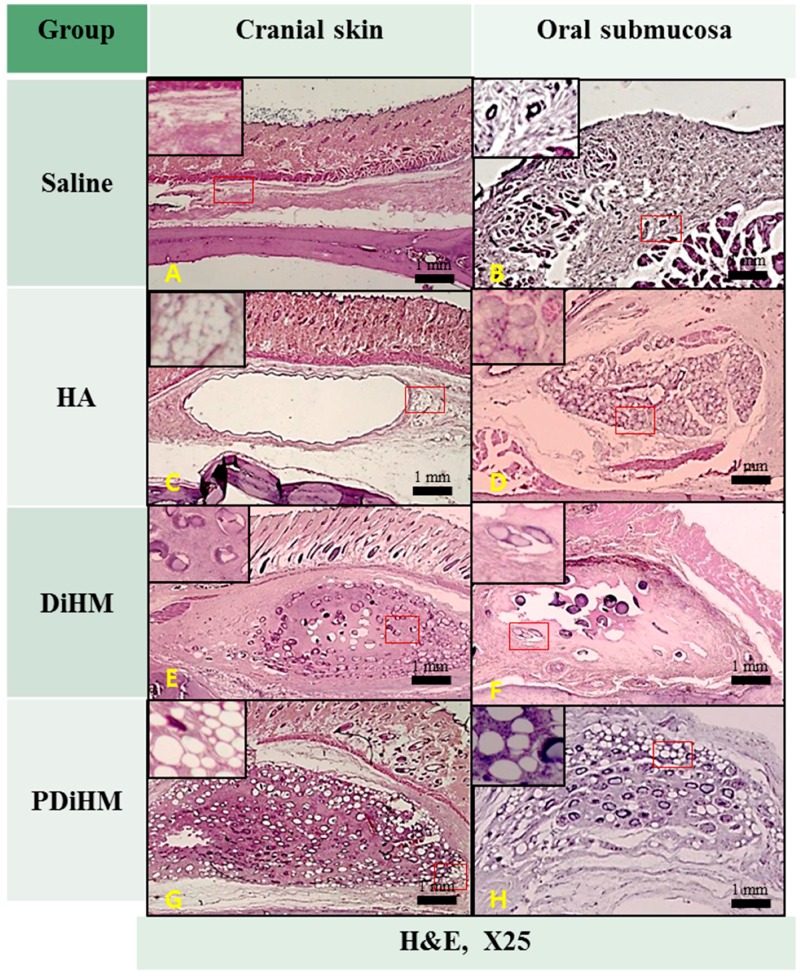
Selected representative specimens were histological features induced by physiological saline (**A**,**B**); HA (**C**,**D**); DiHM (**E**,**F**); and PDiHM (**G**,**H**) of H & E stain at 12 weeks after injection. Hyaluronic acid (HA), cross-linked dextran in hydroxypropyl methylcellulose (DiHM), and cross-linked dextran mixed with PMMA in hydroxypropyl methylcellulose (PDiHM). Scale bar = 1 mm. Red boxes are the areas of enlarge image in left upward of each figure.

**Figure 4:**
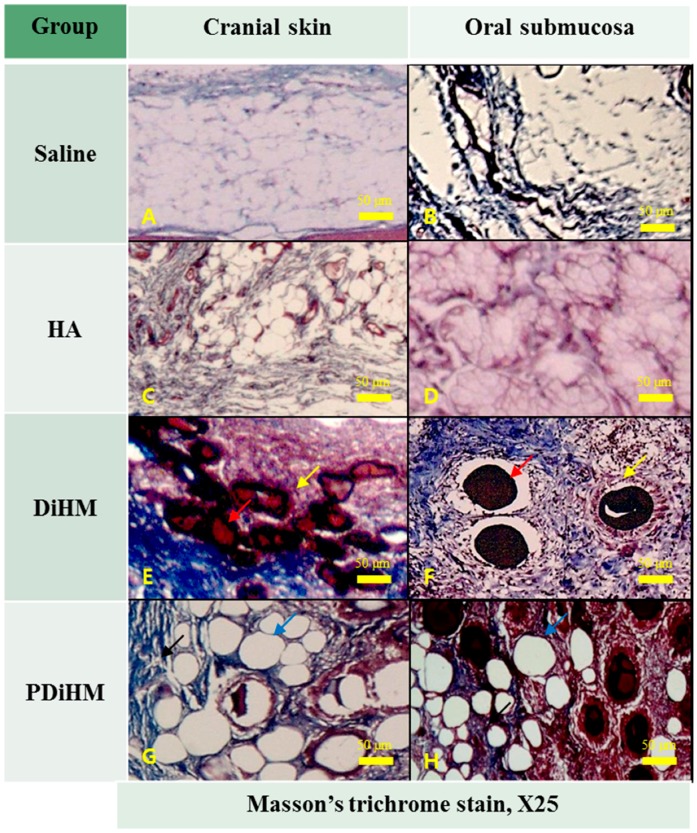
Selected representative specimens were histological features induced by physiological saline (**A**,**B**); HA (**C**,**D**); DiHM (**E**,**F**); and PDiHM (**G**,**H**) of Masson’s trichrome stain at 12 weeks after injection (**E**,**F**); Infiltration of macrophages (yellow arrow) around the dextran microsphere (red arrow) in connective tissue (**G**,**H**); Formation of fibro-connective tissue (black arrow) around the PMMA microspheres (blue arrow). Hyaluronic acid (HA), cross-linked dextran in hydroxypropyl methylcellulose (DiHM), and cross-linked dextran mixed with PMMA in hydroxypropyl methylcellulose (PDiHM). Scale bar = 50 μm.

Skin and submucosal tissues injected with normal saline as a negative control showed no histological abnormality (Figure 3A,B and Figure 4A,B). In the HA group, macrophages and giant cells were rarely detected. HA induced a narrow band of collagen formation, but less than that of DiHM and PDiHM. Macrophages rarely infiltrated into the fibrous connective tissue surrounding HA with minimal neovascularization (Figure 3C,D and Figure 4C,D). In the DiHM group, cross-linked dextran microspheres appeared as red homogeneous material by Masson’s trichrome stain (Figure 4E,F). Substantial fibrous capsules of the fillers were found, and dextran microspheres were surrounded by macrophages. Prominent fibroblast infiltration accompanied by collagen formation was observed among the microspheres. Infiltration of inflammatory cells, some giant cells, and small vessels containing red blood cells was observed in the connective tissue. PDiHM was well maintained, and PMMA microspheres appeared as translucent empty space encapsulated by connective tissue (Figure 4G,H). Moderately thick fibro-connective tissue surrounding the PMMA was observed throughout the experimental period. Macrophages and giant cells were observed around microspheres (Figure 4).

## 3. Discussion

Esthetics has become an important factor in the overall success of most restorations. Consequently, correction of alveolar ridge deformities has been a challenge for dentists, particularly in the esthetic area. Injectable systems can deliver various materials to target locations with minimal invasion, in order to minimize patients’ discomfort and the efficacy can be observed immediately [13].

Hyaluronic acid gel is used widely as a filling material in cosmetic surgery [14,15,16,17,18]. The allergy test is not required because HA is identical in structure among species [19]. However, enzymes such as hyaluronidase and free radicals can rapidly degrade HA polymers [20,21]. In an effort to achieve a longer-lasting effect, DiHM and PDiHM were introduced and recently attracted attention as novel materials for soft tissue augmentation [22,23,24,25,26,27]. Dextran microspheres (diameter: 80–120 μm) are composed of cross-linked dextran molecules [23]. The positive surface charges of dextran microspheres apparently attracted macrophages and released TGF-β and interleukins which stimulate fibroblasts to produce collagen fibers [27]. PMMA is classified as a permanent soft tissue filler. It contains 30–120 μm globular microparticles with a smooth surface, which blocks the phagocytosis of macrophages [28]. It has a long-lasting filler effect and is not biodegraded by the enzyme in living tissue [29]. According to the previous clinical study with PMMA microspheres, it was effective in correction of wrinkles for ten years [30]. Hydroxypropyl methylcellulose, also known as hypromellose, is a semi-synthetic hydrophilic polymer that is widely used as an ophthalmic lubricant and emulsion stabilizer to improve radiologic property [31,32].

The aim of this study is to clinically evaluate intraoral application and effect of novel filler materials compared with hyaluronic acid filler through oral submucosa and cranial subcutaneous injection, using experimental rats. Measurements of both cranial skin and oral mucosa showed a significant augmentation effect compared to the saline group (*p* < 0.001). Sequential size measurements of DiHM and PDiHM showed a superior maintaining ability among the other groups in both skin and oral submucosa during the 12-week experimental period (*p* < 0.001). No statistically significant difference was observed between the DiHM and PDiHM groups. The size of DiHM and PDiHM increased until 4 weeks and tended to decrease later on. By contrast, the HA group exhibited a steady decrease in sequential size in both tissues. In histomorphological examination, HA solution contained few inflammatory cells and induced a narrow band of collagen formation at 12 weeks, while DiHM and PDiHM showed dextran microspheres surrounded by macrophages. In addition, prominent fibroblast infiltration was observed, accompanied by collagen formation. In another study, cross-linked dextran was completely degraded in living tissue within 1–2 years [24]. However, the fibro-connective capsules were not significantly decreased, thus attributing to the formation of collagen for a long time. In PDiHM, PMMA microspheres which appeared as a translucent empty space encapsulated by connective tissue acted as a scaffold and stimulus for constant production of connective tissue. PMMA is not absorbed into the tissues and remains its volume. However, in this study, no statistically significant difference was observed between PDiHM and DiHM. No histological abnormality was found in oral submucosa and cranial skin in all groups. The major host response to the fillers including the formation of fibro-connective tissue, neovascularization, and infiltration of various inflammatory cells, mainly macrophages, was observed in both the DiHM and PDiHM groups.

To the best of our knowledge, this is the only study to evaluate a minimal invasive method for soft tissue augmentation with filler injection. According to the results, DiHM and PDiHM filler materials had no side effect, and their durability was better than that of other well-known HA fillers. Cross-linked dextran microspheres and non-absorbable PMMA are stimulants for encapsulation and scaffolds of collagen formation. Although this study included a small number of subjects and the study period was limited to 12 weeks, it is significant that it is the first study to investigate facial fillers composed of DiHM or PDiHM to oral mucosae. To ensure their effectiveness and safety, additional studies evaluating longer term efficacy with a larger number of subjects will be required.

## 4. Materials and Method

### 4.1. Test Animals and Groups

Fifty 7-week-old Sprague-Dawley male rats were chosen as the experimental animal for this study. They were given solid feed and free access to purified tap water. Regarding the rats’ living environment, the temperature of the breeding room was 22 ± 2 °C, relative humidity 50% ± 15%, light and shade period of 12 h, and intensity of illumination at 150–300 lux. After a week of monitoring, 40 healthy rats were chosen. The samples were divided randomly into 4 groups, with 10 rats included in each group. The weight deviation among groups was within 200 ± 50 g.

### 4.2. Injection of Filler Materials

**Figure 5:**
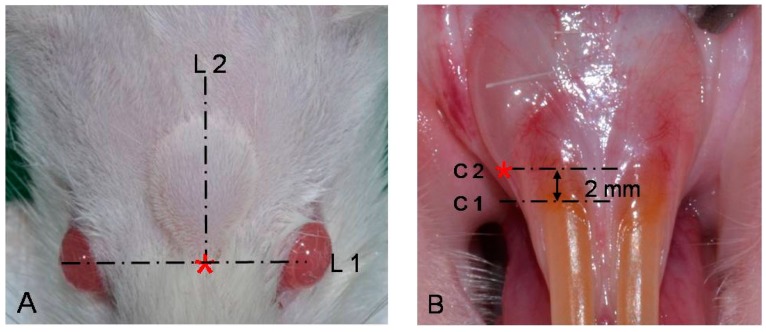
Injection site of cranial skin and in oral submucosa. (**A**) Asterisk (*): injection site, L1: interpupillary line, L2: sagittal line; (**B**) Asterisk (*): injection site, C1: cervical line, C2: 2 mm below the cervical line.

Normal saline solution was administered in the negative control group. HA solution (Restylane^®^, Q-Med AB, Uppsala, Sweden) was administered in the positive control group. The test groups were DiHM (Licol D-1^®^, Chunghwa Medipower Co., Seoul, Korea) and PDiHM (Licol P-1^®^, Chunghwa Medipower Co., Seoul, Korea). Each material was injected into all rats in each group under anesthesia. Cranial subcutaneous injection (0.1 mL) was performed using a 1 mL syringe and 25 G needle. The injection site was the mid-point of the interpupillary line. Intraoral submucosal injection (0.02 mL) was performed using a 0.3 mL syringe and a 25 G needle. The injection site was the ridge crest 2 mm below the cervical line of the mandibular left incisor (Figure 5). The injected areas were disinfected and hair was removed. The experiments were performed according to Jeonnam National University Animal Ethics Committee’s Protocol (CNU IACUC-YB-2013-31) for management and usage of test animals.

### 4.3. Observation of Systemic Signs

During the experimental period, all rats were observed 1 time/day for general signs. When abnormal signs appeared, type, date, and severity were reported individually. In addition, weight change, drink amount, and feed amount were reported every week.

### 4.4. Measurements of Augmentation Area

#### 4.4.1. Photographic Examination for Filler Effect in Oral Submucosa

**Figure 6:**
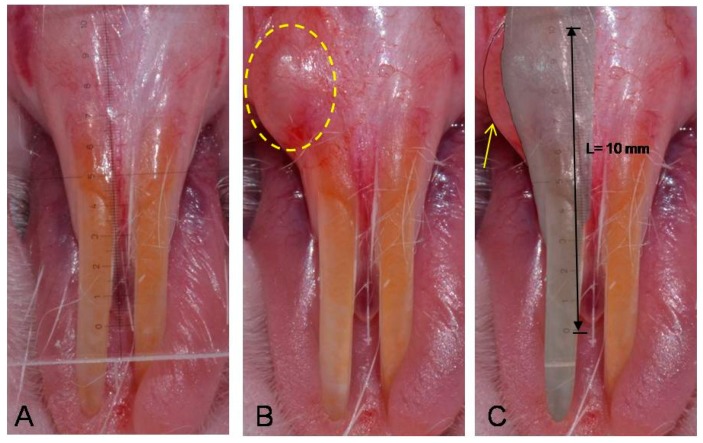
Method for standardizing the measurements of intraoral submucosal injection. (**A**) Initial photo taken with a transparent microscopic ruler before filler injection; (**B**) Photo taken after treatment (circle is the filler injected area); (**C**) The color of the initial photo A was reversed, and overlapped to photo B (arrow indicates augmentation area induced by filler).

Before and immediately after filler injection, intraoral photos were taken for analysis and comparison of the effect of the filler on the injection site. The treated mucosa area of each rat was measured to determine the percentage change from filler injection (baseline) and repeated for 12 weeks in a 4-week term. When taking pictures, general anesthesia was administered by intraperitoneal injection of ketamine 40 mg/kg and xylazine. All images were identified and labeled properly. To standardize the measurement, the camera’s focus was fixed at 1:1 single vision lens setting with a Nikon 3100 digital camera (Nikon Co., Sendai, Japan). Rats were placed on a green cloth under the camera. To minimize potential errors in the measurement such as location and rotation differences, the copy stand, consisting of a plate, was used to position the camera above at a fixed height of 45 cm. The zig was used to immobilize the test samples. When intraoral pictures were taken, a transparent microscopic ruler was positioned adjacent to the site of interest as a reference dimension. The color of pre-injection photos was reversed and overlapped to the photos after treatment. Computer software (i-Solution Inc., Vancouver, BC, Canada) was used to calculate area of the injected sites. Markings of the microscopic ruler in pictures were measured using i-Solution software (INNERVIEW Co., SungNam, Korea), and measurement values of the injected sites were converted to an actual size, using proportional expression (Figure 6).

#### 4.4.2. Radiographic Examination for Filler Effect in Cranial Subcutaneous Tissue

**Figure 7:**
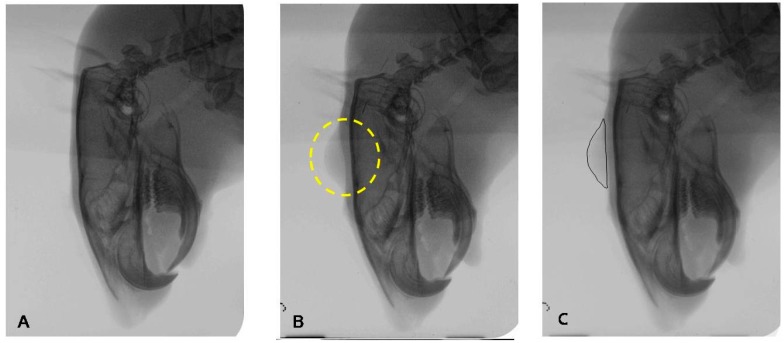
Method for standardizing measurements of cranial subcutaneous injection. (**A**) Initial lateral cephalometric radiograph before filler injection; (**B**) Lateral cephalometric radiograph after treatment (circle is the filler injected area); (**C**) The color of X-ray A was reversed and overlapped to X-ray B (marked circle indicates the augmentation area induced with filler).

Before injection and immediately after injection, lateral cephalometric radiographs of all test animals in each group were taken for radiographic comparison of the filler effect among the test materials. The treated cranial skin area of each rat was measured to determine the percentage change from filler injection (baseline) and repeated for 12 weeks in a 4-week period. The color of the initial x-rays was reversed and overlapped to the X-ray after treatment. Augmentation area was measured using i-Solution software, same as in photographic examination, using the film size as a scale (Figure 7).

#### 4.4.3. Histomorphologic Examination

After 12 weeks from injection of soft tissue filler, all rats in each experimental group were anesthetized with zoletil and suffocated by CO. Dislocation of cervical vertebrae for histotomy was performed. Injected intraoral and cranial connective tissues including the bones of rats were obtained. The tissue was fixed with 10% neutral buffered-formalin for 24 h and dehydrated. It was then embedded in paraffin, and tissue sections were fabricated at a thickness of 4 μm, followed by staining with H & E (hematoxylin and eosin) and Masson’s trichrome stain for observation with a light microscope.

#### 4.4.4. Statistical Analysis

Repeated measure ANOVA was used for comparison of percentage changes of the filler injection area in oral submucosa and cranial skin. If the result did not satisfy Mauchly’s sphericity test, multivariate analysis was performed. ANOVA was performed for comparison of differences within groups at multiple points in measurement time. Duncan’s test was used for *post-hoc* comparison. The level of significance was set at *p* < 0.001. Data analyses were performed using commercially available software (IBM SPSS Statistics 19, SPSS Inc., IBM Co., Armonk, NY, USA).

## 5. Conclusions

In this study on DiHM and PDiHM, no histological abnormality was observed in cranial skin and oral mucosa. DiHM and PDiHM filler materials with injection system provide an excellent alternative surgical method for soft tissue augmentation in oral and craniofacial fields. They can also be widely used in current tissue regenerative fields.
